# FDA-Approved Kinase Inhibitors in Preclinical and Clinical Trials for Neurological Disorders

**DOI:** 10.3390/ph15121546

**Published:** 2022-12-13

**Authors:** Austin Lui, Jordan Vanleuven, David Perekopskiy, Dewey Liu, Desiree Xu, Omar Alzayat, Taiseer Elgokhy, Timothy Do, Meghan Gann, Ryan Martin, Da-Zhi Liu

**Affiliations:** 1 Department of Neurology, University of California at Davis, Davis, CA 95618, USA; 2 Department of Neurological Surgery and Neurology, University of California at Davis, Davis, CA 95618, USA; 3 Mirnova Therapeutics, Inc., Davis, CA 95618, USA

**Keywords:** aberrant cell cycle disease, cancers, neurological disorders, kinase inhibitors

## Abstract

Cancers and neurological disorders are two major types of diseases. We previously developed a new concept termed “Aberrant Cell Cycle Diseases” (ACCD), revealing that these two diseases share a common mechanism of aberrant cell cycle re-entry. The aberrant cell cycle re-entry is manifested as kinase/oncogene activation and tumor suppressor inactivation, which are hallmarks of *both* tumor growth in cancers and neuronal death in neurological disorders. Therefore, some cancer therapies (e.g., kinase inhibition, tumor suppressor elevation) can be leveraged for neurological treatments. The United States Food and Drug Administration (US FDA) has so far approved 74 kinase inhibitors, with numerous other kinase inhibitors in clinical trials, mostly for the treatment of cancers. In contrast, there are dire unmet needs of FDA-approved drugs for neurological treatments, such as Alzheimer’s disease (AD), intracerebral hemorrhage (ICH), ischemic stroke (IS), traumatic brain injury (TBI), and others. In this review, we list these 74 FDA-approved kinase-targeted drugs and identify those that have been reported in preclinical and/or clinical trials for neurological disorders, with a purpose of discussing the feasibility and applicability of leveraging these cancer drugs (FDA-approved kinase inhibitors) for neurological treatments.

## 1. Introduction

**Figure 1:**
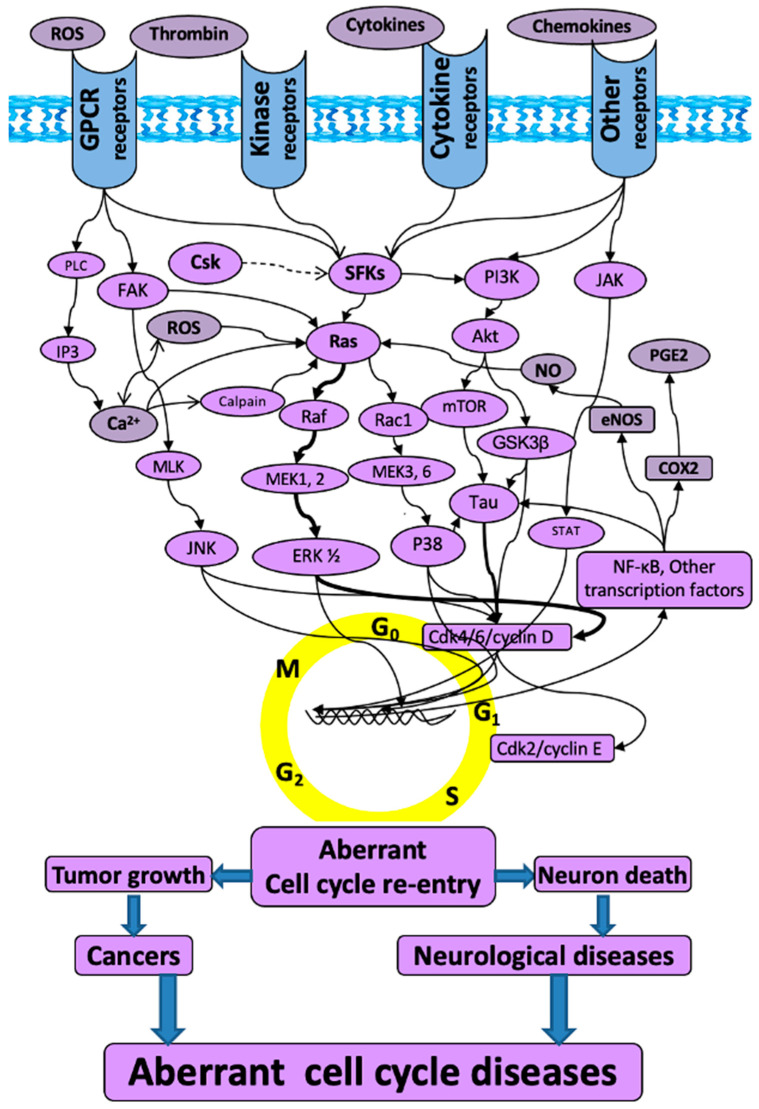
The schematic of “aberrant cell cycle diseases”. The molecules and related mitogenic pathways contributing to the aberrant cell cycle re-entry that is associated with not only tumorigenesis in cancers, but also neuronal death in neurological diseases. The arrows do not necessarily indicate direct binding and/or activation of the downstream molecules; intermediate proteins or kinases may exist. Akt: protein kinase B; Ca^2+^: calcium; Cdk: cyclin-dependent kinase; COX2: cyclooxygenase-2; Csk: c-terminal Src kinase; eNOS: endothelial nitric oxide synthase; ERK: extracellular signal-regulated kinase; FAK: focal adhesion kinase; GPCR: G protein-coupled receptor; GSK3β: glycogen synthase-3 beta; IP3: inositol trisphosphate; JAK: Janus kinase; JNK: c-Jun N-terminal kinases; MEK: mitogen-activated protein kinase kinase; MLK: mixed lineage kinases; mTOR: mammalian target of rapamycin; NF-kB: nuclear factor kappa B; NO: nitric oxide; PGE2: prostaglandin E2; PI3K: phosphatidylinositol 3-kinase; PLC: phospholipase C; Ras: rat sarcoma virus kinase; Rac1: ras-related C3 botulinum toxin substrate 1; Raf: rapidly accelerated fibrosarcoma; ROS: reactive oxygen species; SFKs: Src family kinases; STAT: signal transducer and activator of transcription.

We developed a novel concept of “Aberrant Cell Cycle Disease” (ACCD), revealing that two major types of diseases, cancers and neurological diseases, share the same mechanism of “aberrant cell cycle re-entry” that is manifested as oncogene/kinase activation and/or tumor suppressor inhibition [1]. This concept is an innovation by combining two series of discoveries: (1) tumor cell growth due to aberrant cell cycle re-entry in cancers [2,3,4]; and (2) neuronal death due to aberrant cell cycle re-entry in neurological disorders [5,6,7,8,9,10,11,12,13,14,15,16,17,18]. The ACCD concept itself is novel in two aspects: (1) revealing that cancers and neurological disorders (including TBI) share a common mechanism of aberrant cell cycle re-entry, manifested as kinase/oncoprotein activation and tumor suppressor inactivation [1]; and (2) expanding the key “cell cycle players” from cyclin-dependent kinases (CDKs) and cyclins to Src family kinase (SFK), Jun N-terminal kinase (JNK), extracellular signal-regulated kinase (ERK), and other numerous kinases (Figure 1) [1].

Since kinases are implicated in the process of the cell cycle, kinase inhibitors should be able to block the cell cycle for the treatment of both cancers and neurological disorders. Indeed, compelling evidence shows that a single agent inhibiting the same kinase(s) can treat both cancers and neurological disorders. There is a long list of such agents: CDK inhibitor (roscovitine), SFK inhibitor (PP2), ERK inhibitor (PD98059), ROCK inhibitor (Y-27632), STAT inhibitor (WP1066), mTOR inhibitor (RAD001), and Wnt inhibitor (CWP232291), amongst others. For example, several labs reported that the Src inhibitor PP2 kills cancer cells [19,20,21], while we showed that PP2 protects neurons following acute brain injury in rats [22,23,24].

Following the golden era of cancer drug development in the past few decades, the United States Food and Drug Administration (US FDA) has approved numerous cancer therapies (e.g., RNAi gene therapy, nanoparticle-based in vivo drug delivery reagents, kinase-targeted drugs, and CAR T-cell therapy, as well as others). In contrast, there are very few FDA-approved drugs that benefit patients with certain neurological disorders, such as Alzheimer’s disease (AD), intracerebral hemorrhage (ICH), ischemic stroke (IS), traumatic brain injury (TBI), and other disorders. If state-of-the-art approaches of cancer therapies can be applied to neurological treatments, new breakthroughs will very likely arise in the development of neurological drugs.

The “Aberrant Cell Cycle Disease” concept links cancers and neurological diseases due to their common mechanisms, providing the theoretical framework to leverage cancer elements (e.g., oncogene inhibition) for the treatment of neurological diseases. Since the approval of the first kinase inhibitor (imatinib) in 2001, the US FDA approved a total of 74 kinase inhibitors by the end of 2021, with 12 of these (i.e., tepotinib, umbralisib, idelalisib, duvelisib, copanlisib, alpelisib, tivozanib, trilaciclib, infigratinib, belumosudil, mobocertinib, and asciminib) being approved in 2021.

In this review, we summarize the FDA-approved kinase inhibitors and highlight those that have been tested in experimental models and/or clinical trials for the treatment of neurological disorders, with a purpose of discussing the feasibility and applicability of repurposing these cancer drugs (FDA-approved kinase inhibitors) for the treatment of neurological disorders (e.g., AD, ICH, IS, TBI, and others). Numerous tumor suppressors and non-FDA approved kinase inhibitors are beyond the scope of this review.

## 2. Kinases, Oncoproteins, and Tumor Suppressors

Kinases exist universally in various species, ranging from bacteria to mold to worms and mammals. The human genome encodes more than 500 protein kinases that catalyze various reactions of phosphorylation where high-energy molecules (e.g., ATP) donate phosphate groups to substrate molecules [25,26]. Phosphorylation and its reverse (e.g., dephosphorylation that is catalyzed by phosphatases) are the most frequent post-translational modifications to regulate protein activity. Approximately 13,000 human proteins have phosphorylation sites [27]. Pertaining to their target substrates, human protein kinases are classified as serine–threonine kinases (STK), tyrosine kinases (TK), and dual specificity kinases (STK/TK). Based on the presence or absence of transmembrane receptor structures, TKs can be further divided into receptor TK (RTK) and non-receptor TK (NRTK).

Kinases have predominantly been thought of as oncogenes involved in tumorigenesis [28], since genome-wide studies of kinases have revealed that genetically inherited variants of specific kinases mediate cancer initiation, promotion, and progression, as well as recurrence [29], when they are constitutively overexpressed and/or continuously activated due to chromosomal reshuffling and genetic mutations [29]. However, increasing evidence has shed light on an opposite role for kinases as tumor suppressors. The first identified tumor-suppressing kinase was the protein kinase C (PKC) family members that generally function as tumor suppressors [30]. Subsequently, MKK4 of the mitogen-activated protein kinase kinase (MAPKK) family and DAPK3 of the death-associated protein kinase (DAPK) family were revealed as tumor suppressors, although some controversy still remains [28].

## 3. Neurological Disorder Subtypes and FDA-Approved Drugs for Neurological Treatment

Neurological disorders are diseases of the central and peripheral nervous system. There are more than 600 different neurological disorders, including several main sub-types: (1) acute brain injury, such as ischemic stroke (IS), intracerebral hemorrhage (ICH), subarachnoid hemorrhage (SAH), traumatic brain injury (TBI), spinal cord injury (SCI), epilepsy, and others; (2) neurodegenerative diseases, such as Alzheimer’s disease (AD), Parkinson’s disease (PD), amyotrophic lateral sclerosis (ALS), and others; (3) neurodevelopment diseases, such as autism spectrum disorder (ASD) and cerebral palsy, as well as others; (4) infectious diseases, such as meningitis and encephalitis; and (5) neurological tumors, such as neuroblastoma (NB), glioblastoma (GBM), glioma, and others. Moreover, neurological disorders are often accompanied by mental syndromes, such as when TBI survivors (e.g., 10%–20% of the civilian population and up to 50% of military populations) are subsequently diagnosed with post-traumatic stress disorder (PTSD) [31,32,33], and stroke patients often suffer depression and/or anxiety [34].

These neurological disorders affect hundreds of millions worldwide per year. For example, AD is the most common cause of dementia and represents 60–70% of a total of 47.5 million dementia cases worldwide with 7.7 million new cases every year. The all-cause and all-severity TBIs are estimated to affect ~69 million people each year [35]. More than 6 million people die because of strokes (e.g., IS, ICH) worldwide each year. However, the US FDA has so far approved only one drug (tissue plasminogen activator—tPA) for the treatment of IS and one monoclonal antibody drug (aducanumab) for the treatment of AD, but no drugs have been approved for the treatment of TBI and ICH. In regard to these two FDA-approved neurological drugs, tPA only benefits a small proportion of IS patients, while aducanumab is controversial, as it was approved on the basis that it is capable of reducing a surrogate biomarker, amyloid in the brain, but not on any evidence of clinical benefit. Overall, there are dire unmet needs of effective FDA-approved drugs for the treatment of stroke, TBI, AD, and other neurological disorders.

## 4. FDA-Approved Kinase Inhibitors

Since the approval of the first kinase inhibitor (imatinib) in 2001, the US FDA has so far approved a total of 74 kinase inhibitors, with 12 kinase inhibitors approved in a single year of 2021. Most approved kinase drugs are active against cancers, with a few exceptions for the treatment of non-oncological indications (e.g., tofacitinib for rheumatoid arthritis, sirolimus for organ rejection, nintedanib for idiopathic pulmonary fibrosis). It appears that an increasing number of kinase inhibitors will be approved in the near future, since more than 130 kinase inhibitors were reported to be in Phase-2/3 of clinical trials in 2015 [26]. It is beyond the scope of this review to discuss other protein kinase inhibitors than those approved by FDA.

There are numerous kinase drugs approved for one single indication (Table S1). For example, imatinib, nilotinib, dasatinib, bosutinib, and ponatinib have all been approved for chronic myeloid leukemia (CML). Vandetanib, cabozantinib, and levantinib are used for the treatment for medullary thyroid carcinoma, while imatinib, sunitinib, and regorafenib are indicated also for gastrointestinal stromal tumor (GIST).

Some approved kinase inhibitors have been tested for the treatment of cancer types other than their original indication. For example, abemaciclib, originally approved for the treatment of advanced or metastatic breast cancer in 2015, was recently approved for combination with endocrine therapy (tamoxifen or an aromatase inhibitor) for adjuvant treatment of adult patients with early breast cancer at high risk of recurrence [36]. In addition, some kinase inhibitors have been used in conjunction for certain cancer treatments (e.g., vemurafenib or dabrafenib in combination with trametinib for the treatment of metastatic melanoma) [37].

### 4.1. FDA-Approved Kinase Inhibitors in Clinical Trials for Neurological Disorders

Many of the FDA-approved kinase inhibitors have been tested in clinical and preclinical trials for neurological disorders, though none of them have been approved by the FDA for neurological treatment (Table S1). In terms of clinical trials, Baricitinib, a JAK inhibitor, is being studied in a phase II trial (NCT03921554) along with a phase II and III trial (NCT04517253), for efficacy and safety in Aicardi–Goutieres Syndrome, an inherited encephalopathy that affects infants and usually results in severe mental and physical disabilities.

Bosutinib, an inhibitor of Src and Bcr-Abl, is and has been investigated in clinical trials for different neurodegenerative disorders. There is a phase I trial (NCT04744532) studying the safety and tolerability of bosutinib for amyotrophic lateral sclerosis (ALS), a progressive neurological disease leading to loss of muscle control. The safety, tolerability, and clinical outcomes of bosutinib on patients with dementia with Lewy bodies has also been studied in a completed phase 2 trial (NCT03888222). In preclinical studies, it has been found that bosutinib reduces levels of alpha-synuclein, tau, and beta-amyloid in the CNS, and improves motor and cognitive behavior in animal models [38,39,40]. Bosutinib was also found to promote autophagy and clear protein aggregates in neurons [41,42]. There is also an ongoing phase I trial (NCT02921477) studying the safety and tolerability of bosutinib for mild cognitive impairment (MCI) and dementia.

Cobimetinib, an MEK inhibitor, has been studied in a phase 2 trial (NCT04079179) studying its safety and efficacy in histiocytic disorders, which can lead to neurodegeneration. Dasatinib, an inhibitor of Src, Bcr-Abl, Kit, EGFR, PDGFR, and EPH (EphA2), has been studied in multiple clinical trials examining its effectiveness in treating AD and mild cognitive impairment. Particularly, in four clinical trials (NCT04063124—phase I and II, NCT04785300—phase I and II, NCT04685590- phase II, NCT05422885—phase I/II), the safety, feasibility, and efficacy of dasatinib and quercetin, a flavonoid known to have antioxidant and anti-inflammatory effects, are being assessed.

Everolimus, an inhibitor of mTOR and FKBP, has been extensively evaluated in clinical trials studying different acute brain injury disorders, neurodegenerative disorders, and neurodevelopmental disorders. A phase II trial (NCT03198949) studying the safety and anti-epileptic efficacy of everolimus in patients with Epilepsy and focal cortical dysplasia II, who have failed more than two antiepileptic drugs and surgery, has been recently completed. Everolimus has been shown in animal models to protect seizure-induced brain injury and reduce neuroinflammation associated with seizures [43,44]. A phase II trial (NCT00857259) evaluating the safety and efficacy of everolimus with or without ranibizumab in patients with neovascular age-related macular degeneration, a neurodegenerative disorder that results in a loss of central vision, is currently in progress. Additionally, in a phase I and II trial (NCT02991807), researchers studied whether everolimus can improve neurocognitive outcomes in patients with hamartoma tumor syndrome caused by a PTEN germline mutation. There are also multiple studies (NCT02962414, NCT01730209, NCT01070316, NCT01713946, NCT02451696, NCT01954693, NCT01929642, and NCT012899-12) evaluating the safety and efficacy of everolimus in patients with tuberous sclerosis complex, which is often associated with refractory seizures, cognitive disabilities, autism, focal cortical dysplasia, other neurocognitive problems, and self-injury. Lastly, there is a phase II trial that studied the safety and efficacy of everolimus in patients with seizures who have Sturge–Weber syndrome, a rare disease in which tumors form in the brain (NCT01997255).

Imatinib, a Bcr-Abl, Kit and PDGFR inhibitor, has been studied in several acute brain injury and neurodegenerative disorders. In a phase III trial (NCT03639922), imatinib was studied in ischemic stroke patients to determine whether there was improvement in functional outcomes. Imatinib, administered for 6 days, was added to conventional stroke therapy and started within 8 days of the onset of stroke. Additionally, in a phase II trial (NCT02363361), the safety, uptake, and tolerability of imatinib is being studied in patients with cervical SCI. A phase II trial (NCT03674099) is currently testing imatinib as a novel therapy for multiple sclerosis, comparing its efficacy to methylprednisolone, the standard of care drug for multiple sclerosis relapses. Lastly, imatinib had been studied in a phase I trial (NCT00403156), examining choroidal neovascularization, although this study has been withdrawn.

Nilotinib is a kinase inhibitor that inhibits the activity of Bcr-Abl, PDGFR and DDR1. It has been studied in several neurodegenerative diseases in clinical trials. In a phase I study, nilotinib (NCT03764215) was administered to patients with Huntington disease. Biomarkers, such as phosphorylated tau levels, and functional outcomes were assessed. In an ongoing phase II study (NCT04002674), the use of nilotinib in patients with dementia with Lewy bodies is being studied, particularly on the pharmacokinetics, tolerability, biomarkers, and safety of use. In a phase II study (NCT02947893), the efficacy of nilotinib in AD was studied. Specifically, the effects of nilotinib on cell death was detected with cell markers, and the amyloid concentrations in the brain were assessed with PET scans. Also, a recent phase III clinical trial (NCT05143528) is currently studying the safety and efficacy of nilotinib in patients with early AD using two different dosages. There are also three studies that examined the effects of nilotinib in patients with Parkinson’s disease (NCT02954978, NCT02281474, NCT03205488). In a phase II trial (NCT03932669), the efficacy and adverse events of nilotinib are being studied in patients with spinocerebellar ataxia. In particular, improvement in daily living performance and cerebellar functions are being assessed.

Pazopanib inhibits the activities of VEGFR 1/2/3, PDGFR α/β, FGFR 1/3, Kit, Lck, Fms, and Itk. In terms of neurological disease settings, it is studied mainly in macular degeneration (NCT00659555, NCT01154062, NCT00612456, NCT01072214, NCT00463320, NCT01362348, NCT01134055, NCT01051700, and NCT00733304). Regorafenib, a VEGF kinase inhibitor, was studied in neovascular age-related macular degeneration. After successfully passing phase I clinical trials, (NCT02222207), phase IIa trials were terminated after the results were less effective than the current gold standard treatment [45].

Sirolimus, an mTOR inhibitor initially used as an immunosuppressant in kidney transplants, has been repurposed in a multitude of neurological and psychiatric clinical trials, including cerebral aneurysms (NCT04141020), epilepsy (NCT03646240), Alzheimer’s disease (NCT04629495 and NCT04200911), frontotemporal dementia (NCT04408625), amyotrophic lateral sclerosis (NCT03359538), Parkinson’s disease (NCT04127578), age-related macular degeneration (NCT01445548, NCT00766649, NCT00712491, NCT02357342, NCT02732899, NCT00766337, and NCT00304954), multiple sclerosis (NCT00095329), geographic atrophy associated with age-related macular degeneration (NCT01675947), multiple system atrophy (NCT03589976), Sturge–Weber syndrome (NCT03047980 and NCT02080624), lysosomal diseases (NCT03952637), Leigh syndrome (NCT03747328), tuberous sclerosis complex (NCT04595513, NCT01929642, and NCT05104983), Gaucher disease type 2 (NCT04411654), diabetic retinopathy (NCT00711490), diabetic macular edema (NCT00656643 and NCT00401115), alcohol use disorder (NCT03732248), smoking cessation (NCT04161144), depression (NCT02487485), and stroke prevention (NCT04948749).

Of the 32 clinical trials, two have completed phase I (NCT00401115 and NCT03732248), one has completed phase II/III (NCT03047980), 11 have completed phase I/II or II trials (NCT01445548, NCT00766649, NCT02357342, NCT02732899, NCT00304954, NCT02080624, NCT01929642, NCT00711490, NCT00656643, NCT04161144, and NCT02487485), six have been withdrawn or terminated in phases I/II (NCT00095329, NCT00712491, NCT01675947, NCT03589976, NCT00766337, and NCT03747328), nine are currently in phase I/II or II (NCT04141020, NCT04629495, NCT03359538, NCT04408625, NCT04127578, NCT03952637, NCT04595513, NCT04411654, and NCT05104983), two are in phase I (NCT03646240 and NCT04200911), and one does not specify the phase of the trial (NCT04948749).

Sunitinib is a tyrosine kinase inhibitor, which has been studied in clinical trials to treat both neovascular age-related macular degeneration and diabetic macular edema secondary to retinal vein occlusion. The clinical use of sunitinib for neovascular age-related macular degeneration (NCT03249740) completed phase I clinical trials in 2019. This study tested increasing doses of sunitinib injected intravitreally compared to aflibercept. No data has been published at this time. The use of sunitinib for diabetic macular edema secondary to retinal vein occlusion (NCT04085341) completed phase II trials in 2021. This study specifically looked at the dosing of this compound in patients who had prior treatment with anti-vascular endothelial growth factor.

Temsirolimus, a prodrug of sirolimus and an mTOR inhibitor, has been used in clinical trials for relapsing–remitting multiple sclerosis (NCT00228397). Phase II clinical trials were conducted to assess the long-term tolerability and safety of three different doses of temsirolimus. Tofacitinib, a janus kinase enzyme inhibitor, has been used in three different clinical trials involving neurological disorders: myasthenia gravis, Down syndrome, and depression. Recruiting is underway for an early phase I trial to use tofacitinib in patients with myasthenia gravis (NCT04431895) with the goal to significantly improve quantitative myasthenia gravis scores from a baseline measurement after six months. Currently, a phase II trial using tofacitinib in patients with Down syndrome to treat a multitude of different skin conditions (alopecia areata, atopic dermatitis/eczema, psoriasis, etc.) (NCT04246372) is underway. Lastly, a phase I/II clinical trial comparing tofacitinib to placebo to treat treatment-resistant depression (NCT04141904) had been suspended due to the COVID-19 pandemic.

Trametinib, an MEK inhibitor, is being used in a phase I/II clinical trial for amyotrophic lateral sclerosis (NCT04326283). In this study, researchers will focus on the safety, tolerability, and efficacy of trametinib in ALS patients. Upadacitinib, a selective JAK1 inhibitor, is currently going through phase III trials to treat giant cell arthritis (NCT03725202). In this study, the efficacy of upadacitinib plus corticosteroids is being assessed compared to corticosteroids alone. Finally, a recent phase II trial (NCT05356858) is studying the efficacy and safety of zanubrutinib, a BTK inhibitor, in patients with recurrent neuromyelitis optica spectrum disease, a disease where the immune system damages the optic nerves and spinal cord.

### 4.2. FDA-Approved Kinase Inhibitors in Preclinical Trials for Neurological Disorders

While there have been 16 FDA-approved kinase inhibitors in clinical trials for neurological disorders, there are numerous preclinical studies of FDA-approved kinase inhibitors evaluating their effects on neurological disorders. Abemaciclib has been studied in preclinical models for the treatment of motor neuron degeneration [46] and post-traumatic stress disorder [47]. Afatinib has been tested in preclinical models for the treatment of oxygen/glucose deprivation-induced neuroinflammation [48], multiple sclerosis [49], autoimmune CNS inflammation [49], and nicotine dependance [50]. Axitinib has been tested for treatment of AD [51]. Alectinib has been tested for the potential treatment of binge drinking [52,53]. Baricitinib has been tested in preclinical models for the potential treatment of neurocognitive disorders induced by HIV [54], encephalitis [55,56], multiple sclerosis [56], hypersensitivity in Down syndrome [57], acute spinal cord injury [58], Hutchinson–Gilford progeria [59], and AD [60]. Binimetinib has been shown in a preclinical study to be a potential treatment for some forms of AD [61]. Bosutinib has been tested for the potential treatment of intracerebral hemorrhage [62], cerebral ischemia [63], α-synucleinopathies and tauopathies in neurodegeneration [40,41], Parkinson’s disease [38,42,64], TDP-43 pathology [65], SIN1-mediated neurotoxicity [66], and botulinum neurotoxins [67]. Cabozantinib has been tested for the potential treatment of Rett syndrome [68] and AD [69].

Crizotinib has been tested for the potential treatment of Parkinson’s disease [70], AD [71], persistent pain [72], Toxoplasma gondii (can result in symptoms of congenital neurological and meningoencephalitis) [73], and craniosynostosis associated with Saethre–Chotzen syndrome [74]. Dabrafenib has been tested for the potential treatment of ischemic brain injury [75], spinal cord injury [76], Parkinson’s diseases [77,78], and ataxia caused by neurohistiocytosis of the cerebellum [79]. Dasatinib has been tested for the potential treatment of traumatic brain injury [80], lipopolysaccharide-induced neuroinflammation [81], kainic acid-induced neuroinflammation [82], glaucoma [83], tau-associated pathology [84], multiple sclerosis [85], amyotrophic lateral sclerosis [86,87,88], Parkinson’s disease [87], age-related blood brain barrier dysfunction [89], age-related cognitive dysfunction [89,90], obesity-induced anxiety [91], chronic unpredictable stress-induced cognitive deficits [92], fetal alcohol syndrome [93], and botulinum neurotoxins [67]. Erlotinib has been tested for the potential treatment of nerve fiber injury [94], intracranial aneurysm formation [95], amyotrophic lateral sclerosis [96], diabetic peripheral neuropathy [97,98], and amyloid-β-induced memory loss [99].

Everolimus has been tested for the potential treatment of encephalopathy of prematurity [100], atherosclerosis-associated brain hypoxia [101], ischemic stroke [102,103,104], Alzheimer’s disease [105,106], Huntington disease [107,108], vascular dementia [109], lipopolysaccharide-induced neuroinflammation [110], insulin dysfunction-related cognitive dysfunction [111], glutamate-induced neurotoxicity [112], Guillain–Barre syndrome [113], multiple sclerosis [114], tuberous sclerosis complex-associated autism-like social deficits [115,116], and Lafora disease [117]. Fedratinib has been tested for the potential treatment of ischemic stroke [118], intracerebral hemorrhage [119], Wernicke’s encephalopathy [120,121], and Alzheimer’s disease [122]. Gefitinib has been tested for the potential treatment of spinal cord injury [123], amyloid-β-induced memory loss [99], schizophrenia [124], Streptococcus pneumoniae meningitis [125], and Toxoplasma gondii (can result in symptoms of congenital neurological and meningoencephalitis) [73,126].

Ibrutinib has been tested for the potential treatment of ischemic stroke [127,128], spinal cord injury [129,130], age-related cognitive deterioration [131], Alzheimer’s disease [132,133], lipopolysaccharide-induced neuroinflammation [134], anxiogenic behavior [135], depression [136,137], and cocaine use disorder [138]. Imatinib has been tested for the potential treatment of subarachnoid hemorrhage [139,140,141,142,143,144], intracerebral hemorrhage [145,146,147,148], cerebral small vessel disease [149], traumatic brain injury-induced seizures [150], seizures [150,151], traumatic brain injury [152], ischemia reperfusion-induced cerebral injury [153,154], Alzheimer’s disease [155,156,157,158,159,160,161,162,163,164,165,166,167,168,169,170], Parkinson’s disease [171,172,173,174], prion diseases [175,176,177,178], amyotrophic lateral sclerosis [179], Huntington’s diseases [180], cerebral malaria [181], hypoxic ventilatory depression [182], Niemann–Pick type C disease [183], Niemann–Pick type A disease [184], Gaucher disease [185], simian human immunodeficiency virus encephalitis [186], and morphine tolerance [187]. Lapatinib has been tested for the potential treatment of epileptic seizures [188], organophosphate-induced axonal damage in spinal cord [189], and Alzheimer’s disease [190,191]. Lorlatinib has been tested for the potential treatment of persistent pain [72]. Midostaurin has been tested for the potential treatment of traumatic spinal cord injury [192]. Neratinib has been tested for the potential treatment of AD [193].

Nilotinib has been tested for the potential treatment of epileptic seizures [194], tauopathies [40,41,195], alpha-synucleinopathies [40,42,196,197,198], TDP-43 pathology [64,65], beta-amyloid pathology [195], AD [60,199,200,201], Parkinson’s disease [202,203,204], chorea-acanthocytosis [205,206], and Niemann–Pick type A disease [184]. Palbociclib has been tested for the potential treatment of spinal muscular atrophy [207], amyloid beta-peptide pathology [208], and Parkinson’s disease [209]. Pazopanib has been tested for the potential treatment of tauopathy [210] and neurodegeneration-induced memory and cognitive deficits [211]. Pexidartinib has been tested for the potential treatment of intracerebral hemorrhage [212,213], subarachnoid hemorrhage [214], obesity-related cerebrovascular dysfunction [215], cognitive decline due to brain damage [216], tauopathy [217], AD [218,219], Huntington’s disease [220], multiple sclerosis [221,222,223], spinocerebellar ataxia type 1 [224], Down syndrome [225], peripheral nerve injury-induced mechanical hypersensitivity [226], cocaine addiction [227], and Parkinson’s disease [228]. Ponatinib has been tested for the potential treatment of ischemic stroke [229], epilepsy [230], and cerebral cavernous malformation [231].

Regorafenib has been tested for the potential treatment of AD [232]. Ruxolitinib has been tested for the potential treatment of Parkinson’s disease [233], multiple sclerosis [234,235], Down syndrome [236], cytokine-induced blood brain barrier dysfunction [237], HIV-associated neurocognitive disorders [238], depression-like behaviors and cognitive defects [239], traumatic brain injury [240], ischemic stroke [241], and spinal cord injury [242]. Selumetinib has been tested for the potential treatment of frontotemporal lobar degeneration [243], obsessive-compulsive disorder [244], acrolein-induced neurotoxicity [245], and intracerebral hemorrhage [246].

Sirolimus has been tested for the potential treatment of ischemic stroke [102,247,248,249,250,251,252,253,254,255,256,257,258,259,260,261,262,263,264,265,266,267,268,269,270,271,272,273,274,275,276,277,278], traumatic brain injury [279,280,281,282,283,284,285,286], subarachnoid hemorrhage [287,288,289,290,291,292,293], spinal cord injury [294,295,296,297,298,299,300,301,302,303], germinal matrix hemorrhage [304,305], intracerebral hemorrhage [306,307,308], seizure-induced memory deficits [309,310,311,312], seizure in Leigh syndrome [313], spinal cord ischemia [314,315], preganglionic cervical root transection [316], optic nerve crush [317], alveolar nerve transection [318], ischemic retinal disease [319], multiple sclerosis [320,321,322,323,324,325,326,327,328,329,330,331], Parkinson’s disease [332,333,334,335], cerebral palsy [336], prion disease [337,338,339], AD [340,341,342,343,344], vascular dementia [345], diabetes-induced AD-like pathology [346], diabetes-induced neuropathology [347], Huntington disease [348,349,350,351,352,353], macular degeneration [354], degenerative optic nerve disease [355], retinal neurodegeneration [356], cadmium-induced neurodegeneration [357,358,359,360], spiral ganglion neurons degeneration [361], tauopathy [362,363], synucleinopathy [364,365,366], myasthenia gravis [367,368], iron-induced cognitive impairments [369], intermittent hypoxia-induced cognitive impairments [370], cannabinoid-induced cognitive impairments [371], diabetic perioperative neurocognitive disorders [372], ethanol-induced neurodegeneration [373], aging-related neurodegeneration [374], methylmercury-induced neurotoxicity [375], TDP-43 proteinopathy [376], amyotrophic lateral sclerosis [377], autism spectrum disorders [378,379,380,381,382,383,384], autism-associated behavioral disorders [385], Krabbe disease [386], Down syndrome [387,388,389,390], intellectual disability [391], fetal alcohol spectrum disorders [392,393,394], autism associated with tuberous sclerosis [395,396,397,398], tuberous sclerosis complex [399,400,401], neurodevelopmental defects in tuberous sclerosis complex [402,403], cognitive deficits in tuberous sclerosis complex [404,405], polyhydramnios, megalencephaly, and symptomatic epilepsy syndrome [406], focal cortical dysplasia [407], epilepsy [408,409], epilepsy-induced anxiety and depression [410], Schaaf-Yang syndrome [411], cerebral malaria [412,413,414], neuropathic pain [415], seizure-induced anxiety [416], obesity-induced anxiety and depression [417], mitochondrial encephalopathy [418], diabetes mellitus-related cognitive deficits [419,420,421], nicotine addiction [422], alcohol-related disorders [402,423,424], herpes simplex virus encephalitis [425], depression [426], mania [427], porcine hemagglutinating encephalomyelitis virus [428], anxiety disorders [429,430,431], photochemical damage in retinal photoreceptor cells [432], multisystem proteinopathy [433], NMDA-induced retinal damage [434,435,436], adverse optineurin phenotypes [437], hydrocephalus [438], sleep disorders [439], sepsis-induced cognitive impairment [440], drug-seeking behavior [441,442,443], aging-induced neuroinflammation [444], Koolen–de Vries syndrome [445], TANC2 mutation-induced neuropsychiatric disorders [446], general anesthetic-induced neurodevelopmental disease in fragile-X syndrome [447], Helicobacter pylori-induced depressive and anxiety behavior [448], and age-related hearing loss [449].

Sorafenib has been tested for the potential treatment for subarachnoid hemorrhage [450], ischemic stroke [451], spinal cord injury [452], AD [69,453,454], Parkinson’s disease [455], multiple sclerosis [456,457], rabies [458], Rift Valley fever virus [459], alphaviruses [460], and Picornavirus enterovirus 71 [461]. Sunitinib has been tested for the potential treatment for traumatic brain injury [462], seizure [463], AD [464,465,466], Rett syndrome [68], cognitive impairment associated with HIV [467,468], dengue virus [469], and rabies [470]. Temsirolimus has been tested for the potential treatment for spinal cord injury [300], Parkinson’s disease [471,472], tauopathy [473,474], AD [475], spinocerebellar ataxia type 3 [476], nicotine withdrawal-associated cognitive deficits [477], and X-linked adrenoleukodystrophy [478]. Tofacitinib has been tested for the potential treatment for ischemic stroke [479], AD [480], multiple sclerosis [481,482], Parkinson’s disease [483], amyotrophic lateral sclerosis [484], and Venezuelan equine encephalitis virus [485]. Trametinib has been tested for the potential treatment for traumatic brain injury [486], aneurysmal subarachnoid hemorrhage [487], and brain arteriovenous malformations [488]. Vandetanib has been tested for the potential treatment for germinal matrix hemorrhage [489]. Lastly, Infigratinib has also been tested as a potential treatment for diabetic retinopathy [490].

## 5. Conclusions and Discussions

In summary, there are 16 FDA-approved kinase inhibitors that have been tested in clinical trials for neurological treatments. Since almost all 74 FDA-approved kinase inhibitors have been examined in various animal models of neurological disorders, it appears that more FDA-approved kinase inhibitors will enter clinical trials for neurological treatments in the future. In accordance with the Generics and Biosimilars Initiative, the FDA-approved drugs (including kinase inhibitors) will become available commercially at relatively low prices after expiration of their existing patents. We are optimistic that this repurposing strategy is likely to provide safe, effective, and affordable therapies for neurological disorders.

It is important to note that kinases are also involved in the division of neural stem cells that are associated with neurogenesis and self-repair after brain injury. Therefore, optimization of the dosing regimen of a kinase inhibitor or the combination of a few kinase inhibitors is needed to increase efficacy while reducing side effects, when repurposing the kinase inhibitor(s) to treat neurological disorders.

## Data Availability

Data sharing not applicable.

## Supplementary Materials

The following supporting information can be downloaded at: https://www.mdpi.com/article/10.3390/ph15121546/s1, Table S1: FDA-approved kinase inhibitors in preclinical and clinical trials for neurological disorders.
